# Diethyl 2,6-dihy­droxy-4-(3-nitro­phen­yl)-2,6-bis­(trifluoro­meth­yl)piperidine-3,5-dicarboxyl­ate

**DOI:** 10.1107/S1600536811055346

**Published:** 2012-01-14

**Authors:** Hoong-Kun Fun, Suhana Arshad, B. Palakshi Reddy, V. Vijayakumar, S. Sarveswari

**Affiliations:** a X-ray Crystallography Unit, School of Physics, Universiti Sains Malaysia, 11800 USM, Penang, Malaysia; b Organic Chemistry Division, School of Advanced Sciences, VIT University, Vellore 632 014, India

## Abstract

In the title compound, C_19_H_20_F_6_N_2_O_8_, the eth­oxy and ethyl groups are disordered over two sets of sites, with occupancy ratios of 0.212 (18):0.788 (18) and 0.746 (6):0.254 (6), respectively. The piperidine ring adopts a chair conformation. In the mol­ecule, intra­molecular O—H⋯O hydrogen bonds form two *S*(6) ring motifs. In the crystal, mol­ecules are linked *via* O—H⋯O and C—H⋯O hydrogen bonds, forming dimers.

## Related literature

For studies on 1,4-dihydro­pyridine and piperidones reported by our group, see: Palakshi Reddy *et al.* (2011*a* ▶,*b* ▶,*c* ▶); Rathore *et al.* (2009 ▶); Rajesh *et al.* (2011 ▶). For bond-length data, see: Allen *et al.* (1987 ▶). For hydrogen-bond motifs, see: Bernstein *et al.* (1995 ▶). For ring conformations, see: Cremer & Pople (1975 ▶).
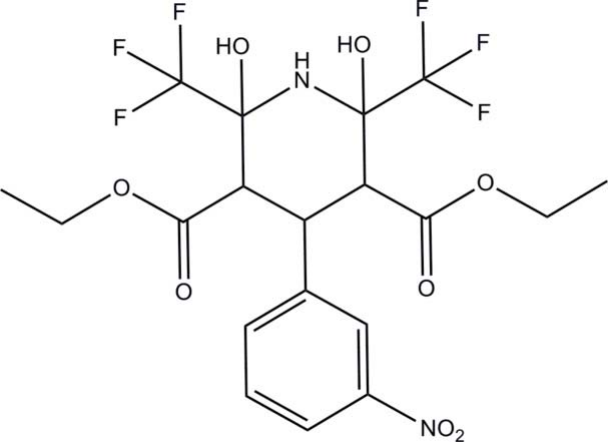


## Experimental

### 

#### Crystal data

C_19_H_20_F_6_N_2_O_8_
*M* *_r_* = 518.37
Monoclinic, 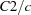

*a* = 17.7353 (16) Å
*b* = 15.2025 (14) Å
*c* = 17.3003 (16) Å
β = 91.049 (2)°
*V* = 4663.7 (7) Å^3^
*Z* = 8
Mo *K*α radiation
μ = 0.14 mm^−1^
*T* = 296 K
0.21 × 0.21 × 0.14 mm

#### Data collection

Bruker SMART APEXII DUO CCD area-detector diffractometer
Absorption correction: multi-scan (*SADABS*; Bruker, 2009 ▶) *T*_min_ = 0.970, *T*_max_ = 0.980
19801 measured reflections
5339 independent reflections
3229 reflections with *I* > 2σ(*I*)
*R*_int_ = 0.028

#### Refinement

*R*[*F* ^2^ > 2σ(*F* ^2^)] = 0.044
*wR*(*F* ^2^) = 0.125
*S* = 1.02
5339 reflections
376 parameters
104 restraints
H atoms treated by a mixture of independent and constrained refinement
Δρ_max_ = 0.21 e Å^−3^
Δρ_min_ = −0.19 e Å^−3^

### 

Data collection: *APEX2* (Bruker, 2009 ▶); cell refinement: *SAINT* (Bruker, 2009 ▶); data reduction: *SAINT*; program(s) used to solve structure: *SHELXTL* (Sheldrick, 2008 ▶); program(s) used to refine structure: *SHELXTL*; molecular graphics: *SHELXTL*; software used to prepare material for publication: *SHELXTL* and *PLATON* (Spek, 2009 ▶).

## Supplementary Material

Crystal structure: contains datablock(s) global, I. DOI: 10.1107/S1600536811055346/rz2690sup1.cif

Structure factors: contains datablock(s) I. DOI: 10.1107/S1600536811055346/rz2690Isup2.hkl

Supplementary material file. DOI: 10.1107/S1600536811055346/rz2690Isup3.cml

Additional supplementary materials:  crystallographic information; 3D view; checkCIF report

## Figures and Tables

**Table 1:** Hydrogen-bond geometry (Å, °)

*D*—H⋯*A*	*D*—H	H⋯*A*	*D*⋯*A*	*D*—H⋯*A*
O7—H1*O*7⋯O6	0.95 (3)	2.06 (3)	2.848 (2)	139 (3)
O8—H1*O*8⋯O4	0.94 (3)	2.09 (3)	2.839 (2)	136 (2)
O7—H1*O*7⋯O4^i^	0.95 (3)	2.28 (3)	2.882 (2)	121 (2)
O8—H1*O*8⋯O6^i^	0.94 (3)	2.26 (3)	2.877 (2)	123 (2)
C11—H11*A*⋯O7^i^	0.93	2.50	3.272 (2)	141
C11—H11*A*⋯O8^i^	0.93	2.44	3.222 (2)	142

Symmetry code: (i) 
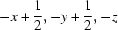
.

**Fig. 1.**
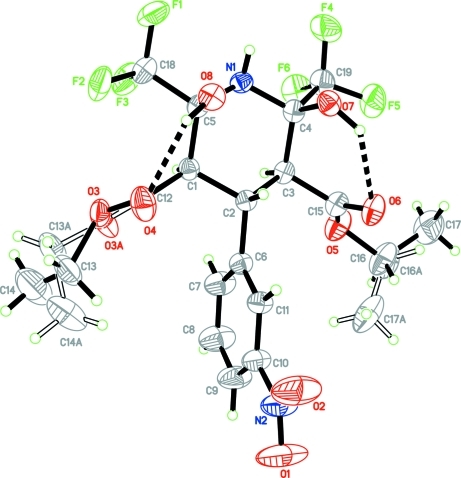
The molecular structure of the title compound, showing 30% probability displacement ellipsoids. All disordered components are shown. Intramolecular hydrogen bonds are shown as dashed lines

**Fig. 2.**
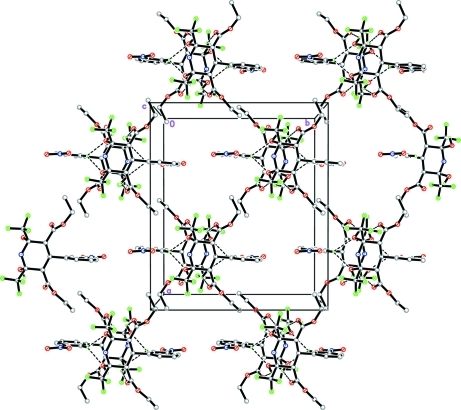
The crystal packing of the title compound viewed along the c axis. Dashed lines represent intermolecular hydrogen bonds. Only the major disordered components are shown.

## supplementary crystallographic information

### Comment

In continuation of our earlier interest in 1,4-DHP's and piperidones (Palakshi Reddy *et al.*, 2011*a*, *b*, *c*; Rathore *et al.*, 2009; Rajesh *et al.*, 2011), herein we report synthesis and crystal structure of diethyl 4-(3-nitrophenyl)-2,6-bis(trifluoromethyl)-1,4-dihydropyridine-3,5-dicarboxylate.

In the molecular structure (Fig. 1), the ethoxy and ethyl groups are disordered over two sets of sites, with 0.212 (18):0.788 (18) and 0.746 (6):0.254 (6) occupancy ratios, respectively. The piperidine ring (N1/C1–C5) adopts a chair conformation with puckering parameters *Q* = 0.5933 (18) Å, θ = 166.58 (17)° and φ = 2.5 (8)° (Cremer & Pople, 1975). In addition, the mean plane through the piperidine ring and the benzene ring (C6–C10) are approximately perpedicular to each other with a dihedral angle of 89.59 (11)°. Intramolecular O7—H1O7···O6 and O8—H1O8···O4 hydrogen bonds (Table 1) stabilize the molecular structure and form two *S*(6) ring motifs (Bernstein *et al.*, 1995). Bond lengths (Allen *et al.*, 1987) and angles are within normal range.

The crystal packing is shown in Fig. 2. The molecules are linked *via* intermolecular O7—H1O7···O4, O8—H1O8···O6, C11—H11A···O7 and C11—H11A···O8 hydrogen bonds (Table 1) to form dimers.

### Experimental

A mixture of 3-nitrobenzaldehyde (1 mmol), ethyl 4,4,4-trifluoro-3-oxobutanoate (2 mmol) and ammonium acetate (1 mmol) were mixed along with 10 ml of ethanol and refluxed for about 2 h. The progress of the reaction was monitored by TLC. After completion, the reaction mixture was cooled to room temperature and allowed to stand for 2 days. The solid product obtained was washed with ether and recrystallized from ethanol to give colourless crystals. M. p.: 395–397 K. Yield: 80%.

### Refinement

The ethoxy and ethyl groups are disordered over two sets of sites, with 0.212 (18):0.788 (18) and 0.746 (6):0.254 (6) occupancy ratios, respectively. Atom H1N1 was located from the difference Fourier map and was fixed at its found location using a riding model with *U*_iso_(H) = 1.5 *U*_eq_(N) [N–H = 0.83 Å]. O-bound H atoms were located in a difference Fourier map and refined freely [O–H = 0.94 (3)–0.95 (3) Å]. The remaining H atoms were positioned geometrically [C–H = 0.93–0.98 Å] and refined using a riding model with *U*_iso_(H) = 1.2 or 1.5 *U*_eq_(C). A rotating group model was applied to the methyl groups. Similarity and rigid bond restraints were used in the final refinement of the disordered groups.

### Crystal data

**Table:**

C_19_H_20_F_6_N_2_O_8_	*F*(000) = 2128
*M**_r_* = 518.37	*D*_x_ = 1.477 Mg m^−^^3^
Monoclinic, *C*2/*c*	Mo *K*α radiation, λ = 0.71073 Å
Hall symbol: -C 2yc	Cell parameters from 4307 reflections
*a* = 17.7353 (16) Å	θ = 2.9–27.1°
*b* = 15.2025 (14) Å	µ = 0.14 mm^−^^1^
*c* = 17.3003 (16) Å	*T* = 296 K
β = 91.049 (2)°	Block, colourless
*V* = 4663.7 (7) Å^3^	0.21 × 0.21 × 0.14 mm
*Z* = 8	

### Data collection

**Table:**

Bruker SMART APEXII DUO CCD area-detector diffractometer	5339 independent reflections
Radiation source: fine-focus sealed tube	3229 reflections with *I* > 2σ(*I*)
graphite	*R*_int_ = 0.028
φ and ω scans	θ_max_ = 27.5°, θ_min_ = 1.8°
Absorption correction: multi-scan (*SADABS*; Bruker, 2009)	*h* = −23→22
*T*_min_ = 0.970, *T*_max_ = 0.980	*k* = −19→19
19801 measured reflections	*l* = −22→22

### Refinement

**Table:**

Refinement on *F*^2^	Secondary atom site location: difference Fourier map
Least-squares matrix: full	Hydrogen site location: inferred from neighbouring sites
*R*[*F*^2^ > 2σ(*F*^2^)] = 0.044	H atoms treated by a mixture of independent and constrained refinement
*wR*(*F*^2^) = 0.125	*w* = 1/[σ^2^(*F*_o_^2^) + (0.0493*P*)^2^ + 2.0652*P*] where *P* = (*F*_o_^2^ + 2*F*_c_^2^)/3
*S* = 1.02	(Δ/σ)_max_ < 0.001
5339 reflections	Δρ_max_ = 0.21 e Å^−^^3^
376 parameters	Δρ_min_ = −0.19 e Å^−^^3^
104 restraints	Extinction correction: *SHELXTL* (Sheldrick, 2008), Fc^*^=kFc[1+0.001xFc^2^λ^3^/sin(2θ)]^-1/4^
Primary atom site location: structure-invariant direct methods	Extinction coefficient: 0.00084 (15)

### Special details

**Table:**

Geometry. All e.s.d.'s (except the e.s.d. in the dihedral angle between two l.s. planes) are estimated using the full covariance matrix. The cell e.s.d.'s are taken into account individually in the estimation of e.s.d.'s in distances, angles and torsion angles; correlations between e.s.d.'s in cell parameters are only used when they are defined by crystal symmetry. An approximate (isotropic) treatment of cell e.s.d.'s is used for estimating e.s.d.'s involving l.s. planes.
Refinement. Refinement of *F*^2^ against ALL reflections. The weighted *R*-factor *wR* and goodness of fit *S* are based on *F*^2^, conventional *R*-factors *R* are based on *F*, with *F* set to zero for negative *F*^2^. The threshold expression of *F*^2^ > σ(*F*^2^) is used only for calculating *R*-factors(gt) *etc*. and is not relevant to the choice of reflections for refinement. *R*-factors based on *F*^2^ are statistically about twice as large as those based on *F*, and *R*- factors based on ALL data will be even larger.

### Fractional atomic coordinates and isotropic or equivalent isotropic displacement parameters (Å^2^)

**Table:**

	*x*	*y*	*z*	*U*_iso_*/*U*_eq_	Occ. (<1)
F1	0.36213 (8)	0.39434 (9)	0.23934 (8)	0.0892 (5)	
F2	0.44369 (7)	0.30673 (10)	0.19217 (8)	0.0896 (5)	
F3	0.37694 (9)	0.26642 (10)	0.28772 (7)	0.0906 (5)	
F4	0.10958 (8)	0.36187 (10)	0.23426 (8)	0.0903 (5)	
F5	0.04642 (7)	0.25576 (11)	0.18300 (8)	0.0940 (5)	
F6	0.11954 (8)	0.23302 (10)	0.28156 (7)	0.0887 (5)	
O1	0.26864 (19)	−0.15284 (15)	−0.12905 (13)	0.1564 (11)	
O2	0.27330 (16)	−0.01793 (15)	−0.15351 (11)	0.1287 (9)	
O3	0.4418 (19)	0.106 (3)	0.163 (2)	0.060 (4)	0.212 (18)
C13	0.4982 (16)	0.046 (2)	0.1072 (17)	0.103 (5)	0.212 (18)
H13A	0.4682	0.0137	0.0696	0.123*	0.212 (18)
H13B	0.5317	0.0851	0.0795	0.123*	0.212 (18)
C14	0.5328 (18)	−0.001 (2)	0.145 (2)	0.146 (8)	0.212 (18)
H14A	0.5785	−0.0162	0.1188	0.219*	0.212 (18)
H14B	0.5040	−0.0539	0.1532	0.219*	0.212 (18)
H14C	0.5449	0.0258	0.1934	0.219*	0.212 (18)
O3A	0.4362 (6)	0.0929 (8)	0.1583 (7)	0.085 (2)	0.788 (18)
C13A	0.5125 (4)	0.0683 (6)	0.1339 (4)	0.0949 (19)	0.788 (18)
H13C	0.5483	0.0687	0.1770	0.114*	0.788 (18)
H13D	0.5305	0.1067	0.0934	0.114*	0.788 (18)
C14A	0.4973 (5)	−0.0272 (5)	0.1033 (5)	0.127 (3)	0.788 (18)
H14D	0.5434	−0.0518	0.0846	0.191*	0.788 (18)
H14E	0.4604	−0.0253	0.0621	0.191*	0.788 (18)
H14F	0.4789	−0.0630	0.1446	0.191*	0.788 (18)
O4	0.41966 (8)	0.17953 (11)	0.05490 (9)	0.0736 (5)	
O6	0.09483 (8)	0.14011 (10)	0.04494 (8)	0.0658 (4)	
N1	0.24395 (8)	0.29412 (10)	0.19612 (8)	0.0462 (4)	
H1N1	0.2380	0.3481	0.1947	0.069*	
N2	0.27191 (15)	−0.07751 (16)	−0.10851 (13)	0.0909 (7)	
O7	0.16066 (8)	0.30333 (9)	0.09062 (8)	0.0552 (4)	
O8	0.32528 (8)	0.32280 (9)	0.09458 (8)	0.0551 (4)	
C1	0.32495 (9)	0.17525 (12)	0.15245 (10)	0.0447 (4)	
H1A	0.3228	0.1466	0.2031	0.054*	
C2	0.25852 (9)	0.14162 (11)	0.10170 (9)	0.0413 (4)	
H2A	0.2559	0.1780	0.0549	0.050*	
C3	0.18628 (10)	0.15865 (12)	0.14797 (10)	0.0455 (4)	
H3A	0.1924	0.1309	0.1988	0.055*	
C4	0.17731 (10)	0.25802 (12)	0.16000 (10)	0.0471 (4)	
C5	0.31612 (10)	0.27463 (12)	0.16372 (10)	0.0458 (4)	
C6	0.26648 (11)	0.04661 (12)	0.07686 (10)	0.0480 (4)	
C7	0.27170 (15)	−0.02213 (14)	0.12943 (13)	0.0756 (7)	
H7A	0.2712	−0.0101	0.1821	0.091*	
C8	0.27757 (19)	−0.10787 (16)	0.10466 (16)	0.0975 (10)	
H8A	0.2809	−0.1532	0.1406	0.117*	
C9	0.27849 (17)	−0.12666 (15)	0.02689 (16)	0.0892 (8)	
H9A	0.2826	−0.1844	0.0097	0.107*	
C10	0.27314 (13)	−0.05851 (14)	−0.02482 (12)	0.0649 (6)	
C11	0.26732 (11)	0.02741 (13)	−0.00142 (11)	0.0525 (5)	
H11A	0.2640	0.0724	−0.0378	0.063*	
C12	0.39930 (11)	0.15197 (14)	0.11582 (12)	0.0543 (5)	
C15	0.11874 (11)	0.11741 (14)	0.10727 (11)	0.0530 (5)	
O5	0.09056 (9)	0.05249 (11)	0.14855 (9)	0.0774 (5)	
C16	0.0290 (7)	0.0034 (8)	0.1123 (8)	0.088 (2)	0.746 (6)
H16A	0.0284	−0.0558	0.1331	0.105*	0.746 (6)
H16B	0.0375	−0.0007	0.0572	0.105*	0.746 (6)
C17	−0.0461 (2)	0.0461 (3)	0.1254 (3)	0.1125 (18)	0.746 (6)
H17A	−0.0856	0.0084	0.1062	0.169*	0.746 (6)
H17B	−0.0483	0.1014	0.0987	0.169*	0.746 (6)
H17C	−0.0524	0.0558	0.1798	0.169*	0.746 (6)
C16A	0.020 (2)	−0.006 (3)	0.121 (3)	0.110 (7)	0.254 (6)
H16C	−0.0045	−0.0335	0.1640	0.132*	0.254 (6)
H16D	−0.0160	0.0283	0.0908	0.132*	0.254 (6)
C17A	0.0617 (8)	−0.0715 (10)	0.0718 (8)	0.129 (6)	0.254 (6)
H17D	0.0261	−0.1119	0.0490	0.194*	0.254 (6)
H17E	0.0973	−0.1033	0.1035	0.194*	0.254 (6)
H17F	0.0879	−0.0411	0.0317	0.194*	0.254 (6)
C18	0.37467 (12)	0.31059 (15)	0.22174 (12)	0.0631 (6)	
C19	0.11297 (13)	0.27698 (17)	0.21556 (12)	0.0662 (6)	
H1O7	0.1249 (18)	0.270 (2)	0.0612 (18)	0.131 (12)*	
H1O8	0.3669 (16)	0.3016 (18)	0.0676 (16)	0.107 (10)*	

### Atomic displacement parameters (Å^2^)

**Table:**

	*U*^11^	*U*^22^	*U*^33^	*U*^12^	*U*^13^	*U*^23^
F1	0.1027 (11)	0.0731 (9)	0.0911 (10)	−0.0184 (8)	−0.0143 (8)	−0.0273 (8)
F2	0.0528 (8)	0.1192 (12)	0.0964 (10)	−0.0196 (7)	−0.0136 (7)	−0.0125 (9)
F3	0.1094 (11)	0.1064 (11)	0.0548 (7)	−0.0210 (9)	−0.0313 (7)	0.0068 (7)
F4	0.0893 (10)	0.0979 (11)	0.0846 (9)	0.0251 (8)	0.0223 (7)	−0.0234 (8)
F5	0.0472 (7)	0.1460 (14)	0.0895 (10)	0.0020 (8)	0.0177 (7)	−0.0190 (9)
F6	0.0921 (10)	0.1193 (12)	0.0559 (8)	0.0020 (8)	0.0331 (7)	0.0065 (8)
O1	0.278 (4)	0.0839 (15)	0.1069 (17)	0.0169 (18)	−0.0065 (19)	−0.0513 (13)
O2	0.235 (3)	0.0943 (16)	0.0572 (11)	0.0125 (16)	0.0104 (14)	−0.0131 (11)
O3	0.044 (7)	0.079 (8)	0.057 (7)	0.004 (6)	−0.012 (6)	−0.002 (6)
C13	0.079 (8)	0.125 (10)	0.103 (10)	0.054 (8)	−0.009 (8)	−0.031 (9)
C14	0.138 (15)	0.166 (17)	0.132 (16)	0.080 (13)	−0.004 (13)	−0.017 (14)
O3A	0.073 (4)	0.105 (5)	0.078 (3)	0.048 (3)	0.006 (3)	0.011 (3)
C13A	0.078 (3)	0.126 (5)	0.080 (3)	0.052 (3)	−0.007 (2)	−0.012 (3)
C14A	0.142 (6)	0.118 (5)	0.123 (5)	0.060 (4)	0.038 (4)	0.009 (4)
O4	0.0514 (9)	0.1103 (13)	0.0595 (9)	0.0113 (8)	0.0097 (7)	0.0095 (9)
O6	0.0560 (9)	0.0897 (11)	0.0515 (8)	−0.0178 (7)	−0.0057 (7)	0.0130 (8)
N1	0.0508 (9)	0.0483 (9)	0.0396 (8)	−0.0002 (7)	0.0042 (7)	−0.0060 (7)
N2	0.132 (2)	0.0692 (15)	0.0711 (14)	0.0120 (13)	0.0019 (13)	−0.0252 (12)
O7	0.0592 (8)	0.0592 (9)	0.0471 (7)	0.0066 (7)	−0.0009 (6)	0.0057 (6)
O8	0.0608 (9)	0.0571 (8)	0.0475 (7)	−0.0029 (7)	0.0057 (6)	0.0089 (6)
C1	0.0440 (10)	0.0502 (10)	0.0399 (9)	0.0018 (8)	−0.0015 (7)	0.0017 (8)
C2	0.0446 (10)	0.0419 (10)	0.0375 (9)	−0.0009 (8)	0.0014 (7)	0.0024 (7)
C3	0.0443 (10)	0.0533 (11)	0.0389 (9)	−0.0062 (8)	0.0027 (7)	0.0050 (8)
C4	0.0442 (10)	0.0583 (12)	0.0391 (9)	−0.0002 (8)	0.0062 (8)	−0.0001 (8)
C5	0.0466 (10)	0.0516 (11)	0.0392 (9)	−0.0041 (8)	0.0007 (8)	0.0000 (8)
C6	0.0562 (11)	0.0420 (10)	0.0456 (10)	−0.0019 (8)	0.0001 (8)	0.0043 (8)
C7	0.120 (2)	0.0524 (13)	0.0545 (12)	0.0009 (13)	−0.0073 (12)	0.0081 (11)
C8	0.167 (3)	0.0501 (14)	0.0751 (17)	0.0019 (16)	−0.0128 (18)	0.0135 (13)
C9	0.136 (2)	0.0401 (12)	0.0913 (19)	0.0018 (14)	−0.0111 (17)	−0.0053 (13)
C10	0.0848 (16)	0.0515 (12)	0.0583 (12)	0.0006 (11)	−0.0009 (11)	−0.0111 (10)
C11	0.0652 (13)	0.0460 (11)	0.0462 (10)	0.0023 (9)	0.0020 (9)	0.0001 (9)
C12	0.0459 (11)	0.0676 (13)	0.0494 (11)	0.0056 (9)	−0.0035 (9)	−0.0047 (10)
C15	0.0502 (11)	0.0625 (13)	0.0466 (11)	−0.0102 (9)	0.0058 (9)	0.0052 (9)
O5	0.0769 (11)	0.0878 (11)	0.0671 (9)	−0.0391 (9)	−0.0074 (8)	0.0215 (8)
C16	0.082 (4)	0.095 (4)	0.086 (4)	−0.056 (3)	−0.011 (3)	0.019 (3)
C17	0.075 (3)	0.134 (4)	0.129 (4)	−0.032 (3)	0.012 (2)	−0.007 (3)
C16A	0.101 (14)	0.120 (13)	0.109 (12)	−0.031 (12)	−0.013 (10)	0.037 (10)
C17A	0.136 (11)	0.134 (12)	0.116 (10)	−0.064 (9)	−0.050 (8)	0.012 (8)
C18	0.0622 (14)	0.0700 (15)	0.0568 (12)	−0.0126 (11)	−0.0081 (10)	−0.0028 (11)
C19	0.0577 (13)	0.0868 (17)	0.0546 (12)	0.0053 (12)	0.0142 (10)	−0.0058 (12)

### Geometric parameters (Å, °)

**Table:**

F1—C18	1.329 (3)	C1—C2	1.544 (2)
F2—C18	1.337 (2)	C1—H1A	0.9800
F3—C18	1.324 (2)	C2—C6	1.514 (2)
F4—C19	1.332 (3)	C2—C3	1.545 (2)
F5—C19	1.338 (3)	C2—H2A	0.9800
F6—C19	1.326 (3)	C3—C15	1.514 (3)
O1—N2	1.200 (3)	C3—C4	1.534 (3)
O2—N2	1.195 (3)	C3—H3A	0.9800
O3—C12	1.31 (4)	C4—C19	1.533 (3)
O3—C13	1.67 (5)	C5—C18	1.532 (3)
C13—C14	1.14 (5)	C6—C11	1.386 (2)
C13—H13A	0.9700	C6—C7	1.387 (3)
C13—H13B	0.9700	C7—C8	1.377 (3)
C14—H14A	0.9600	C7—H7A	0.9300
C14—H14B	0.9600	C8—C9	1.376 (3)
C14—H14C	0.9600	C8—H8A	0.9300
O3A—C12	1.326 (12)	C9—C10	1.371 (3)
O3A—C13A	1.473 (13)	C9—H9A	0.9300
C13A—C14A	1.567 (18)	C10—C11	1.372 (3)
C13A—H13C	0.9700	C11—H11A	0.9300
C13A—H13D	0.9700	C15—O5	1.322 (2)
C14A—H14D	0.9600	O5—C16	1.454 (11)
C14A—H14E	0.9600	O5—C16A	1.59 (4)
C14A—H14F	0.9600	C16—C17	1.503 (14)
O4—C12	1.196 (2)	C16—H16A	0.9700
O6—C15	1.202 (2)	C16—H16B	0.9700
N1—C4	1.436 (2)	C17—H17A	0.9600
N1—C5	1.438 (2)	C17—H17B	0.9600
N1—H1N1	0.8269	C17—H17C	0.9600
N2—C10	1.476 (3)	C16A—C17A	1.51 (4)
O7—C4	1.410 (2)	C16A—H16C	0.9700
O7—H1O7	0.95 (3)	C16A—H16D	0.9700
O8—C5	1.414 (2)	C17A—H17D	0.9600
O8—H1O8	0.94 (3)	C17A—H17E	0.9600
C1—C12	1.515 (3)	C17A—H17F	0.9600
C1—C5	1.532 (3)		
			
C12—O3—C13	106 (3)	C11—C6—C7	118.70 (18)
C14—C13—O3	110 (3)	C11—C6—C2	118.75 (16)
C14—C13—H13A	111.0	C7—C6—C2	122.54 (17)
O3—C13—H13A	109.7	C8—C7—C6	120.9 (2)
C14—C13—H13B	109.7	C8—C7—H7A	119.5
O3—C13—H13B	109.7	C6—C7—H7A	119.5
H13A—C13—H13B	108.2	C9—C8—C7	120.2 (2)
C12—O3A—C13A	117.4 (10)	C9—C8—H8A	119.9
O3A—C13A—C14A	100.3 (9)	C7—C8—H8A	119.9
O3A—C13A—H13C	111.7	C10—C9—C8	118.6 (2)
C14A—C13A—H13C	111.7	C10—C9—H9A	120.7
O3A—C13A—H13D	111.7	C8—C9—H9A	120.7
C14A—C13A—H13D	111.7	C9—C10—C11	122.1 (2)
H13C—C13A—H13D	109.5	C9—C10—N2	119.5 (2)
C13A—C14A—H14D	109.5	C11—C10—N2	118.4 (2)
C13A—C14A—H14E	109.5	C10—C11—C6	119.42 (18)
H14D—C14A—H14E	109.5	C10—C11—H11A	120.3
C13A—C14A—H14F	109.5	C6—C11—H11A	120.3
H14D—C14A—H14F	109.5	O4—C12—O3	123.9 (19)
H14E—C14A—H14F	109.5	O4—C12—O3A	124.9 (6)
C4—N1—C5	118.89 (14)	O4—C12—C1	124.46 (18)
C4—N1—H1N1	105.3	O3—C12—C1	111.2 (19)
C5—N1—H1N1	107.8	O3A—C12—C1	110.6 (6)
O2—N2—O1	122.1 (2)	O6—C15—O5	124.63 (18)
O2—N2—C10	119.4 (2)	O6—C15—C3	124.15 (17)
O1—N2—C10	118.5 (3)	O5—C15—C3	111.21 (16)
C4—O7—H1O7	109.0 (19)	C15—O5—C16	116.0 (6)
C5—O8—H1O8	110.3 (17)	C15—O5—C16A	123.6 (17)
C12—C1—C5	112.05 (15)	O5—C16—C17	112.0 (8)
C12—C1—C2	110.23 (14)	O5—C16—H16A	109.2
C5—C1—C2	108.68 (14)	C17—C16—H16A	109.2
C12—C1—H1A	108.6	O5—C16—H16B	109.2
C5—C1—H1A	108.6	C17—C16—H16B	109.2
C2—C1—H1A	108.6	H16A—C16—H16B	107.9
C6—C2—C1	113.77 (14)	C17A—C16A—O5	99 (2)
C6—C2—C3	112.92 (14)	C17A—C16A—H16C	112.0
C1—C2—C3	106.34 (13)	O5—C16A—H16C	112.0
C6—C2—H2A	107.9	C17A—C16A—H16D	112.0
C1—C2—H2A	107.9	O5—C16A—H16D	112.0
C3—C2—H2A	107.9	H16C—C16A—H16D	109.7
C15—C3—C4	112.82 (16)	C16A—C17A—H17D	109.5
C15—C3—C2	110.25 (15)	C16A—C17A—H17E	109.5
C4—C3—C2	108.93 (14)	H17D—C17A—H17E	109.5
C15—C3—H3A	108.2	C16A—C17A—H17F	109.5
C4—C3—H3A	108.2	H17D—C17A—H17F	109.5
C2—C3—H3A	108.2	H17E—C17A—H17F	109.5
O7—C4—N1	109.97 (15)	F3—C18—F1	106.91 (18)
O7—C4—C19	107.16 (15)	F3—C18—F2	107.10 (19)
N1—C4—C19	105.75 (15)	F1—C18—F2	106.74 (18)
O7—C4—C3	112.72 (14)	F3—C18—C5	113.17 (17)
N1—C4—C3	110.43 (15)	F1—C18—C5	112.19 (18)
C19—C4—C3	110.53 (16)	F2—C18—C5	110.38 (17)
O8—C5—N1	109.87 (15)	F6—C19—F4	106.42 (18)
O8—C5—C1	112.92 (14)	F6—C19—F5	107.68 (19)
N1—C5—C1	110.29 (14)	F4—C19—F5	107.00 (19)
O8—C5—C18	106.40 (15)	F6—C19—C4	112.99 (18)
N1—C5—C18	105.58 (15)	F4—C19—C4	111.84 (19)
C1—C5—C18	111.45 (16)	F5—C19—C4	110.60 (17)
			
C12—O3—C13—C14	−174 (4)	C7—C6—C11—C10	0.1 (3)
C12—O3A—C13A—C14A	−108.6 (8)	C2—C6—C11—C10	−178.85 (18)
C12—C1—C2—C6	48.7 (2)	C13—O3—C12—O4	−33 (3)
C5—C1—C2—C6	171.81 (14)	C13—O3—C12—O3A	66 (13)
C12—C1—C2—C3	173.59 (15)	C13—O3—C12—C1	153.9 (18)
C5—C1—C2—C3	−63.26 (17)	C13A—O3A—C12—O4	8.6 (12)
C6—C2—C3—C15	−47.4 (2)	C13A—O3A—C12—O3	−80 (14)
C1—C2—C3—C15	−172.88 (15)	C13A—O3A—C12—C1	−175.2 (8)
C6—C2—C3—C4	−171.73 (14)	C5—C1—C12—O4	−57.3 (3)
C1—C2—C3—C4	62.81 (17)	C2—C1—C12—O4	63.8 (3)
C5—N1—C4—O7	−74.3 (2)	C5—C1—C12—O3	115.9 (16)
C5—N1—C4—C19	170.28 (16)	C2—C1—C12—O3	−123.0 (16)
C5—N1—C4—C3	50.7 (2)	C5—C1—C12—O3A	126.5 (5)
C15—C3—C4—O7	−54.4 (2)	C2—C1—C12—O3A	−112.3 (5)
C2—C3—C4—O7	68.37 (18)	C4—C3—C15—O6	57.1 (3)
C15—C3—C4—N1	−177.85 (14)	C2—C3—C15—O6	−65.0 (3)
C2—C3—C4—N1	−55.08 (18)	C4—C3—C15—O5	−124.01 (18)
C15—C3—C4—C19	65.5 (2)	C2—C3—C15—O5	113.95 (18)
C2—C3—C4—C19	−171.74 (15)	O6—C15—O5—C16	3.6 (7)
C4—N1—C5—O8	73.9 (2)	C3—C15—O5—C16	−175.3 (6)
C4—N1—C5—C1	−51.2 (2)	O6—C15—O5—C16A	0(2)
C4—N1—C5—C18	−171.71 (16)	C3—C15—O5—C16A	−179 (2)
C12—C1—C5—O8	54.7 (2)	C15—O5—C16—C17	−85.9 (8)
C2—C1—C5—O8	−67.35 (18)	C16A—O5—C16—C17	74 (16)
C12—C1—C5—N1	178.06 (14)	C15—O5—C16A—C17A	84 (3)
C2—C1—C5—N1	56.01 (18)	C16—O5—C16A—C17A	63 (15)
C12—C1—C5—C18	−65.0 (2)	O8—C5—C18—F3	−174.73 (17)
C2—C1—C5—C18	172.94 (15)	N1—C5—C18—F3	68.5 (2)
C1—C2—C6—C11	−120.73 (19)	C1—C5—C18—F3	−51.2 (2)
C3—C2—C6—C11	117.93 (19)	O8—C5—C18—F1	64.2 (2)
C1—C2—C6—C7	60.4 (2)	N1—C5—C18—F1	−52.5 (2)
C3—C2—C6—C7	−61.0 (2)	C1—C5—C18—F1	−172.31 (16)
C11—C6—C7—C8	0.0 (4)	O8—C5—C18—F2	−54.7 (2)
C2—C6—C7—C8	178.9 (2)	N1—C5—C18—F2	−171.46 (17)
C6—C7—C8—C9	0.1 (5)	C1—C5—C18—F2	68.8 (2)
C7—C8—C9—C10	−0.3 (5)	O7—C4—C19—F6	175.74 (18)
C8—C9—C10—C11	0.3 (4)	N1—C4—C19—F6	−67.0 (2)
C8—C9—C10—N2	−178.3 (3)	C3—C4—C19—F6	52.6 (2)
O2—N2—C10—C9	−174.1 (3)	O7—C4—C19—F4	−64.2 (2)
O1—N2—C10—C9	6.8 (4)	N1—C4—C19—F4	53.1 (2)
O2—N2—C10—C11	7.2 (4)	C3—C4—C19—F4	172.61 (17)
O1—N2—C10—C11	−171.9 (3)	O7—C4—C19—F5	55.0 (2)
C9—C10—C11—C6	−0.2 (4)	N1—C4—C19—F5	172.24 (18)
N2—C10—C11—C6	178.4 (2)	C3—C4—C19—F5	−68.2 (2)

### Hydrogen-bond geometry (Å, °)

**Table:**

*D*—H···*A*	*D*—H	H···*A*	*D*···*A*	*D*—H···*A*
O7—H1O7···O6	0.95 (3)	2.06 (3)	2.848 (2)	139 (3)
O8—H1O8···O4	0.94 (3)	2.09 (3)	2.839 (2)	136 (2)
O7—H1O7···O4^i^	0.95 (3)	2.28 (3)	2.882 (2)	121 (2)
O8—H1O8···O6^i^	0.94 (3)	2.26 (3)	2.877 (2)	123 (2)
C11—H11A···O7^i^	0.93	2.50	3.272 (2)	141
C11—H11A···O8^i^	0.93	2.44	3.222 (2)	142

Symmetry codes: (i) −*x*+1/2, −*y*+1/2, −*z*.
